# Evaluation of a Clinical Decision Support Strategy to Increase Seasonal Influenza Vaccination Among Hospitalized Children Before Inpatient Discharge

**DOI:** 10.1001/jamanetworkopen.2021.17809

**Published:** 2021-07-22

**Authors:** Evan W. Orenstein, Omar ElSayed-Ali, Swaminathan Kandaswamy, Erin Masterson, Reena Blanco, Pareen Shah, Patricia Lantis, Amy Kolwaite, Thomas E. Dawson, Edwin Ray, Christy Bryant, Srikant Iyer, Andi L. Shane, Stephanie Jernigan

**Affiliations:** 1 Department of Pediatrics, Emory University School of Medicine, Atlanta, Georgia; 2 Division of Hospital Medicine, Children’s Healthcare of Atlanta, Atlanta, Georgia; 3 Information Services and Technology, Children’s Healthcare of Atlanta, Atlanta, Georgia; 4 Department of Pediatrics, Washington University in St Louis, St Louis, Missouri; 5 Division of Emergency Medicine, Children’s Healthcare of Atlanta, Atlanta, Georgia; 6 Nell Hodgson Woodruff School of Nursing, Emory University, Atlanta, Georgia; 7 Division of Infectious Diseases, Children’s Healthcare of Atlanta, Atlanta, Georgia; 8 Division of Nephrology, Children’s Healthcare of Atlanta, Atlanta, Georgia

## Abstract

**Importance:**

Hospitalized children are at increased risk of influenza-related complications, yet influenza vaccine coverage remains low among this group. Evidence-based strategies about vaccination of vulnerable children during all health care visits are especially important during the COVID-19 pandemic.

**Objective:**

To design and evaluate a clinical decision support (CDS) strategy to increase the proportion of eligible hospitalized children who receive a seasonal influenza vaccine prior to inpatient discharge.

**Design, Setting, and Participants:**

This quality improvement study was conducted among children eligible for the seasonal influenza vaccine who were hospitalized in a tertiary pediatric health system providing care to more than half a million patients annually in 3 hospitals. The study used a sequential crossover design from control to intervention and compared hospitalizations in the intervention group (2019-2020 season with the use of an intervention order set) with concurrent controls (2019-2020 season without use of an intervention order set) and historical controls (2018-2019 season with use of an order set that underwent intervention during the 2019-2020 season).

**Interventions:**

A CDS intervention was developed through a user-centered design process, including (1) placing a default influenza vaccine order into admission order sets for eligible patients, (2) a script to offer the vaccine using a presumptive strategy, and (3) just-in-time education for clinicians addressing vaccine eligibility in the influenza order group with links to further reference material. The intervention was rolled out in a stepwise fashion during the 2019-2020 influenza season.

**Main Outcomes and Measures:**

Proportion of eligible hospitalizations in which 1 or more influenza vaccines were administered prior to discharge.

**Results:**

Among 17 740 hospitalizations (9295 boys [52%]), the mean (SD) age was 8.0 (6.0) years, and the patients were predominantly Black (n = 8943 [50%]) or White (n = 7559 [43%]) and mostly had public insurance (n = 11 274 [64%]). There were 10 997 hospitalizations eligible for the influenza vaccine in the 2019-2020 season. Of these, 5449 (50%) were in the intervention group, and 5548 (50%) were concurrent controls. There were 6743 eligible hospitalizations in 2018-2019 that served as historical controls. Vaccine administration rates were 31% (n = 1676) in the intervention group, 19% (n = 1051) in concurrent controls, and 14% (n = 912) in historical controls (*P* < .001). In adjusted analyses, the odds of receiving the influenza vaccine were 3.25 (95% CI, 2.94-3.59) times higher in the intervention group and 1.28 (95% CI, 1.15-1.42) times higher in concurrent controls than in historical controls.

**Conclusions and Relevance:**

This quality improvement study suggests that user-centered CDS may be associated with significantly improved influenza vaccination rates among hospitalized children. Stepwise implementation of CDS interventions was a practical method that was used to increase quality improvement rigor through comparison with historical and concurrent controls.

## Key Points

**Question:**

Is a clinical decision support (CDS) strategy associated with improved influenza vaccination rates before discharge among eligible hospitalized children?

**Findings:**

In this quality improvement study, the combinination of a default-checked influenza vaccine order in admission order sets for eligible patients with a nursing script using a presumptive strategy to offer the vaccine was associated with significantly higher odds of the hospitalized child receiving the influenza vaccine compared with concurrent and historical controls.

**Meaning:**

This study suggests that a user-centered CDS strategy may improve vaccination rates among vulnerable, hospitalized children.

## Teaser

This quality improvement study evaluates a clinical decision support strategy to increase the proportion of eligible hospitalized children who receive a seasonal influenza vaccine prior to inpatient discharge.

## Introduction

Influenza vaccination prevents millions of health care visits and thousands of influenza-related deaths every year.^1,2^ Despite its effectiveness, vaccine coverage rates were only 63% in US children in the 2018-2019 influenza season.^3^ Hospitalized children often have high-risk conditions predisposing them to influenza morbidity and mortality, yet influenza vaccine coverage rates in this group range from 33% to 59% in published studies, lower than the national average.^4,5,6^ Children from lower-income households are more likely to be hospitalized and are less likely to have access to medical homes,^7,8^ which is associated with similar discrepancies in other routine vaccinations.^9,10^ Most children admitted to a hospital with influenza have had 1 or more missed opportunities for vaccination prior to their hospitalization.^4^ The Centers for Disease Control and Prevention Advisory Committee on Immunization Practices recommends vaccination during hospitalizations in addition to routine health care visits.^11^ However, influenza vaccination status is ascertained less often at acute-care visits and is often not considered a high priority.^12^ The importance of identifying strategies to identify and vaccinate vulnerable children has increased in the setting of the COVID-19 pandemic.^13^

Several studies describe strategies to improve influenza vaccine administration among hospitalized children.^6,14^ Pollack et al^5^ demonstrated that a nursing-focused screening tool that was triggered for eligible patients and that placed an influenza vaccine order in the background of the patient’s medical record significantly improved influenza vaccine administration rates (adjusted odds ratio [OR], 6.77; 95% CI, 6.14-7.47). However, the absolute vaccination rate increased only from 2.1% to 8.0%, leaving substantial room for improvement. Rao et al^15^ found that a weekly, manually generated email reminder to clinical teams indicating the influenza vaccination status of each patient improved ordering practices. In a subsequent study, Rao et al^16^ expanded on this intervention to include educational modules, huddles, and reminders for nurses as well as vaccination lists in the electronic health record (EHR) and financial incentives for residents; this expansion was associated with a 1.39 times higher odds of a child being vaccinated against influenza before discharge. Freedman et al^17^ incorporated influenza vaccine orders into oncology admission order sets along with family education and clinic staff reports of vaccine uptake in outpatient settings. Their combined efforts were associated with increased influenza immunization rates among oncology patients from 20% to 65%. A quality improvement study of children with asthma admitted to a pediatric hospital medicine service combining a nurse-driven protocol with an alert for physicians, changes to the asthma history and physical note template, and substantial educational efforts was associated with increased influenza immunization rates from 13% to 57% over 4 years.^6^ However, most of these interventions required ongoing maintenance, and the association of each intervention bundle element with the outcome was unclear.

Communication about vaccines using presumptive communication strategies is known to improve influenza vaccine uptake in ambulatory settings.^18,19^ Alerts for influenza vaccine eligibility with a default order have also modestly improved vaccine uptake in primary care settings, although the effect size may not be sufficient owing to workflow issues and problems addressing clinician and patient acceptance.^20,21^ However, it remains unknown whether these strategies are used in hospital settings, what their effectiveness is at promoting influenza vaccine uptake, or what the necessary adaptations are for the inpatient setting.

Clinical decision support (CDS) embedded in the EHR can deliver patient-specific recommendations to encourage evidence-based practices. However, the association of CDS with process measures and patient outcomes has been inconsistent,^22^ particularly in promoting health maintenance interventions in acute care settings.^23,24,25,26^ A review of 35 studies found that the association of CDS with promoting inpatient pneumococcal vaccine in adults varied widely.^27^ A qualitative study of physicians’ desired characteristics for influenza vaccine alerts noted that CDS needed to show up early during a patient visit, automatically identify patients eligible for the influenza vaccine, facilitate vaccine ordering, and generate appropriate documentation.^28^ We hypothesize that a CDS system incorporating these characteristics and iteratively designed through formative usability testing with frontline clinicians would improve influenza vaccination rates among eligible hospitalized children. The aim of this study was to increase the proportion of eligible hospitalized children who receive the influenza vaccine prior to discharge.

## Methods

### Ethical Considerations and Reporting Guidelines

This study was determined to be non–human participants research as a local quality improvement study by the Children’s Healthcare of Atlanta institutional review board. This study is reported according to the Standards for Quality Improvement Reporting Excellence (SQUIRE) 2.0 reporting guideline for quality improvement reports and the Safety-related EHR Research (SAFER) Reporting Framework for safety-related EHR interventions.^29,30^

### Setting

This study was performed in an urban pediatric health system including 3 freestanding children’s hospitals: 1 academic tertiary care center (hospital A), 1 community tertiary care center (hospital B), and 1 academic secondary care center (hospital C). Pediatric residents are frequently involved in inpatient care at hospitals A and C but less often at hospital B. All 3 sites are live on a common EHR instance of Epic Systems. Influenza vaccines were available at all 3 sites generally from September through April of each year. At the beginning of influenza season each year, a special grand rounds was devoted to reviewing influenza burden, new guidelines, and promoting the influenza vaccine.

Prior to the intervention, a noninterruptive alert in the discharge navigator appeared for patients aged 6 months or older with no record of influenza vaccine in the local database or state immunization registry for the current influenza season (eFigure 1 in the Supplement). During the baseline period (September 1, 2018, to May 1, 2019), this alert was ignored 94% of the time that it appeared (23 632 of 25 276), with an influenza vaccine order placed only 2% of the time (409 of 25 276). Of the 1609 influenza vaccine orders placed in the baseline period, 409 (25%) were placed through this noninterruptive alert, with the rest placed as ad hoc orders.

### Interventions

We performed a user and task analysis^31^ through informal interviews of key stakeholders in the influenza vaccination process, including general medical ward nurses, pediatric residents, pediatric hospital medicine attending physicians, and pharmacists, to identify barriers to vaccine administration and inform a suite of EHR interventions addressing those barriers (eTable 1 in the Supplement). Patients and families were not interviewed in the development of this intervention.

#### Nursing Admission Questionnaire Adjustments

As part of the admission process, nursing staff members ask a series of questions of all parents. In the immunization section of the nursing administration questionnaire, we added questions with scripting designed to present the influenza vaccine using a presumptive communication strategy (eFigure 2B in the Supplement).

#### Influenza Vaccine Order Group

We developed an order group including a default-checked influenza vaccine order automatically timed for 12 pm the day after admission, just-in-time education regarding influenza vaccine appropriateness for specific populations (eg, patients receiving corticosteroids, patients with asthma exacerbations, patients with egg allergy, patients with cancer, and other immunocompromised patients), as well as links to the state immunization registry, Centers for Disease Control and Prevention guidance, and supporting literature (eFigure 2A in the Supplement).^32,33,34^ The order group would dynamically appear if the patient met the following criteria: (1) aged 6 months or older, (2) no influenza vaccine in our local EHR system or the state immunization registry for the current influenza season, (3) no history of anaphylaxis to any influenza vaccine in the local EHR system, and (4) no documentation by nursing staff indicating that the patient has already received influenza vaccine, has had an anaphylactic reaction, or parental refusal (see the Nursing Admission Questionnaire Adjustments subsection). If the nursing admission questionnaire was not filled out when the clinician accessed the orders and all other eligibility criteria were met, then the default-checked influenza vaccine order would show up in the admission order set. The clinician could manually unselect the influenza vaccine order from the order group if they chose. The order group was added sequentially to specific admission order sets as described in the Study of the Interventions subsection. The order group did not appear for patients 8 years of age or younger who had received 1 dose of influenza vaccine in the current season but had not received the influenza vaccine in any prior season and therefore should receive a second dose at least 4 weeks after the first dose.

#### Communication Tip Sheet

Our influenza vaccine working group, including infectious diseases specialists, worked with our marketing and parent and family advocacy groups to develop a tip sheet focused on (1) the benefits associated with a presumptive method for introducing vaccines^18,35,36^; (2) responses to common issues raised by families, such as “my kids have never had the flu,” “I got the flu shot once and it gave me the flu,” or “[the influenza vaccine] doesn’t work”; and (3) vaccine facts, such as adverse effects and time to protection. We included a link to this tip sheet in the influenza vaccine order, which also appeared in the medication administration activity for nursing staff, but it was not immediately available in the nursing admission questionnaire.

A description of all 3 interventions was provided at educational sessions for nurses and residents and at an EHR physician oversight committee meeting prior to implementation. It was reviewed again with clinical department leaders prior to each stepwise implementation.

### Interventions

**Figure 1.**
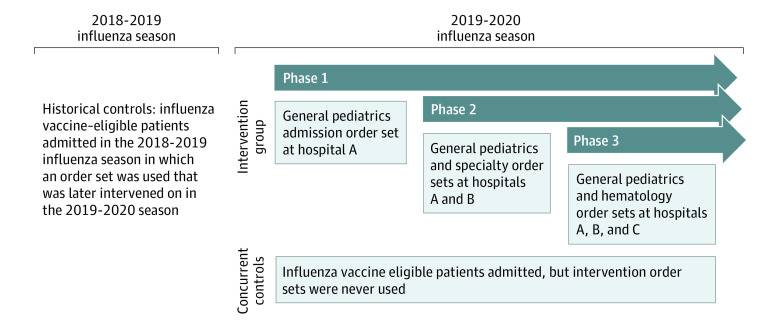
Study Design With Sequential Crossover From Control to Intervention

We implemented the influenza vaccine order group using a sequential crossover design from control to intervention by clustering at the order set level (Figure 1; eTable 2 in the Supplement). On September 19, 2019, the order group was added to the general pediatrics admission order set restricted to hospital A only (phase 1). Two weeks later on October 3, 2019, the same order group was added to the general pediatrics admission order set at hospital B; it was also added to other commonly used disease-specific and general admission order sets on the general pediatrics, critical care, hematology and oncology, gastroenterology, and neurology services at hospitals A and B (phase 2A). On October 29, 2019, the order group was added to additional order sets for the pulmonary, gastroenterology, and hematology and oncology services at hospitals A and B (phase 2B). Finally, on November 19, 2019, the order group was added to general pediatrics and hematology admission order sets at hospital C (phase 3). Although the plan for sequential expansion was devised by the investigators, the actual order of expansion was not randomized or predetermined by the investigators. Rather, the order group was implemented initially on the general pediatrics service at hospital A for convenience (because this was the clinical practice area for one of us [E.W.O.]), and then pilot data were emailed to clinical leaders of each hospital service. Subsequent prioritization of the intervention rollout was based on email responsiveness, the chance to demonstrate the intervention and pilot data to clinical leaders, and consideration of constraints from the information technology department. The nursing admission questionnaire adjustments and communication tip sheet were implemented on September 19, 2019, across the health system.

We defined eligible hospitalization with the first 3 criteria used for the influenza vaccine order group (see the Interventions subsection). To keep the cohort consistent between the seasons, hospitalizations were included even if the nurse documented that the patient had already received the vaccine elsewhere or the parent refused the vaccine because this documentation was performed inconsistently in the 2018-2019 season. We classified each eligible hospitalization as part of the intervention group if at least 1 order was placed using an order set that included the influenza order group (although this order did not have to be for the influenza vaccine). Concurrent controls were defined as eligible hospitalizations during the 2019-2020 influenza season (September 1, 2019, to May 1, 2020) in which no orders were placed from any order set that included the influenza order group. Historical controls were defined as eligible hospitalizations during the 2018-2019 influenza season (September 1, 2018, to May 1, 2019) in which at least 1 order was placed using an order set that subsequently underwent an intervention during the following influenza season—for example, if a clinician placed admission orders using the general pediatrics admission order set in the 2018-2019 influenza season (which, at the time, did not contain the influenza vaccine order group), then that hospitalized patient would count as a historical control.

Race/ethnicity was assessed in this study to look for disparities in vaccine uptake. Race/ethnicity was assigned based on what was documented in the EHR, which is generally based on patient and family self-report from a set of discrete choices during the registration process. The number of pediatric complex chronic conditions^37,38^ was determined from active diagnoses in the problem list in the EHR at the time of discharge.

### Measures

Our primary outcome measure was the proportion of eligible hospitalizations with at least 1 dose of influenza vaccine administered prior to discharge. Our process measure was the proportion of eligible hospitalizations in which an influenza vaccine order was placed prior to discharge.

### Statistical Analysis

Demographic characteristics and outcome, process, and balance measures were compared between the intervention group, concurrent controls, and historical controls. Continuous variables with normal distribution were compared across all 3 intervention groups using 1-way analysis of variance and nonnormal variables with the Kruskal-Wallis test. Categorical variables were compared using the χ^2^ test. The null hypothesis for these tests was that the distribution of the dependent variable was the same across all 3 intervention groups. All *P* values were from 2-sided tests and results were deemed statistically significant at *P* < .05.

In addition, we evaluated the difference in influenza vaccine administration rates among eligible hospitalizations in the intervention group, concurrent control group, and historical control group visually using run charts and analytically using mixed-effects logistic regression adjusting for age, sex, race/ethnicity, insurance status, and week of the influenza season. The unit of analysis was the hospitalization, so patient and hospital were added as random effects to account for individual patients with multiple hospitalizations during the influenza season as well as workflow differences between the hospitals. Statistical analyses were performed using R, version 3.6.1 (R Group for Statistical Computing) with the lme4 package.^39,40^

## Results

Among 17 740 hospitalizations (9295 boys [52%]), the mean (SD) age was 8.0 (6.0) years, and the patients were predominantly Black (n = 8943 [50%]) or White (n = 7559 [43%]) and mostly had public insurance (n = 11 274 [64%]) (Table 1). In the 2019-2020 influenza season, there were 10 997 hospitalizations meeting the eligibility criteria. An order set containing the dynamic influenza vaccine order group was used for 5449 patients (50%) composing the intervention group, leaving 5548 hospitalizations (50%) in the concurrent control group. In the 2018-2019 influenza season, there were 6743 eligible hospitalizations after nursing screening in which an order was placed using an order set that subsequently underwent an intervention—these hospitalizations composed the historical control group. The intervention group and the historical control group had a significantly greater proportion of female, Black, and non-Hispanic patients, as well as patients with public insurance, compared with the concurrent control group, although the magnitude of these differences was not large. The intervention group had fewer patients with complex chronic conditions (n = 1699 [31%]) compared with the historical control group (n = 2372 [35%]; *P* < .001) and the concurrent control group (n = 2038 [37%]; *P* < .001 compared with the intervention group and *P* = .08 compared with historical control group).

**Table 1.** Demographic Characteristics and Outcome, Process, and Balance Measures of Patients in the Intervention, Concurrent Control, and Historical Control Groups

Characteristic	Hospitalizations, No. (%)			*P* value^a^
	Intervention group (n = 5449)	Concurrent control group (n = 5548)	Historical control group (n = 6743)	
Demographic characteristics				
Age				
Mean (SD), y	8.1 (5.9)	8.0 (6.1)	8.0 (5.9)	.50
6-23 mo	901 (19)	1242 (25)	1340 (20)	<.001
2-4 y	1118 (23)	929 (19)	1359 (20)	
5-12 y	1561 (32)	1452 (29)	2200 (33)	
13-17 y	1066 (22)	1178 (23)	1441 (22)	
≥18 y	207 (4)	219 (4)	292 (4)	
Sex				
Female	2721 (50)	2617 (47)	3107 (46)	.001
Male	2728 (50)	2931 (53)	3636 (54)	
Race^b^				
Black	2836 (52)	2442 (44)	3665 (54)	<.001
White	2223 (41)	2682 (48)	2654 (39)	<.001
Asian	164 (3)	178 (3)	202 (3)	.76
Other^c^	32 (1)	36 (1)	34 (1)	.90
Unknown	406 (8)	458 (8)	487 (7)	.08
Ethnicity				
Non-Hispanic	4703 (86)	4700 (85)	5913 (88)	<.001
Hispanic	730 (13)	829 (15)	807 (12)	<.001
Unknown	16 (0.3)	19 (0.3)	23 (0.3)	.87
Insurance^d^				
Public	3493 (64)	3377 (61)	4404 (65)	<.001
Private	1757 (32)	1996 (36)	2084 (31)	<.001
Self-pay	199 (4)	175 (3)	255 (4)	.15
CCCs				
Mean (SD), No.	0.89 (1.80)	1.19 (2.18)	1.03 (1.87)	<.001^a^
≥1 CCCs	1699 (31)	2038 (37)	2372 (35)	<.001
Outcome measure				
Vaccine administered	1676 (31)	1051 (19)	912 (14)	<.001
Process measure				
Vaccine ordered	4199 (77)	1488 (27)	1024 (15)	<.001

Abbreviation: CCCs, complex chronic conditions.

^a^ Age and mean number of CCCs were compared between the 3 groups using 1-way analysis of variance. All other comparisons were completed using the χ^2^ test of independence. The null hypothesis for these tests was that the distribution of the dependent variable was the same across the intervention, concurrent control, and historical control groups.

^b^ More than 1 race was documented for some encounters, so the numbers do not add to 100%.

^c^ Other race included patients for whom all options selected were among the following: American Indian or Alaska Native, Native Hawaiian or other Pacific Islander, other, and other or declined.

^d^ More than 1 insurance status was documented for some encounters, so the numbers do not add to 100%.

**Figure 2.**
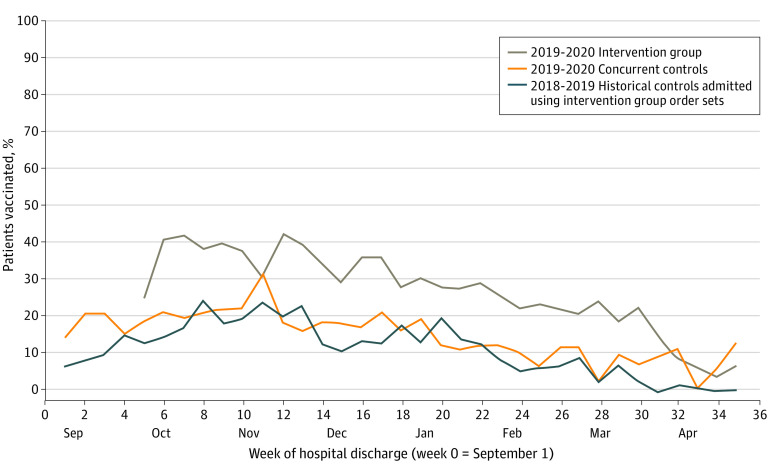
Run Chart of Influenza Vaccine Administration Rates Stratified by Intervention, Concurrent Control, and Historical Control Group

The influenza vaccine was ordered and administered most frequently in the intervention group, followed by the concurrent control group and the historical control group (Figure 2). Of the 5449 hospitalizations in the intervention group, an influenza vaccine was ordered for 4199 hospitalizations (77%), and the vaccine was administered during 1676 hospitalizations (31%). In the concurrent control group, the influenza vaccine was ordered for 1488 hospitalizations (27%; *P* < .001 compared with the intervention group) and administered during 1051 hospitalizations (19%; *P* < .001 compared with the intervention group). Among historical controls, the vaccine was ordered for 1024 hospitalizations (15%; *P* < .001 compared with both the intervention and concurrent control groups) and administered during 912 hospitalizations (14%; *P* < .001 compared with both the intervention and concurrent control groups).

In univariate analysis, vaccine administration was most strongly associated with the intervention group (crude OR, 3.10; 95% CI, 2.81-3.41) and was also associated with the concurrent control group (crude OR, 1.56; 95% CI, 1.41-1.72) compared with historical controls (Table 2). Vaccine administration was also significantly more common earlier in the influenza season, among age groups outside 13 to 17 years, for patients of unknown race, for Hispanic patients, and for those with public insurance.

**Table 2.** Factors Associated With Influenza Vaccination Among Hospitalized Children

Factor	Odds ratio (95% CI)	
	Crude	Adjusted
Intervention characteristics		
Historical control group	1 [Reference]	1 [Reference]
Concurrent control group	1.56 (1.41-1.72)	1.28 (1.15-1.42)
Intervention group	3.10 (2.81-3.41)	3.25 (2.94-3.59)
Week of influenza season (per week)	0.97 (0.97-0.98)	0.96 (0.96-0.97)
Demographic characteristics		
Age group		
6-23 mo	1 [Reference]	1 [Reference]
2-4 y	1.11 (0.99-1.24)	1.04 (0.93-1.17)
5-12 y	1.01 (0.91-1.13)	0.99 (0.88-1.10)
13-17 y	0.72 (0.64-0.82)	0.72 (0.64-0.81)
≥18 y	0.95 (0.78-1.16)	0.98 (0.80-1.21)
Sex		
Female	1 [Reference]	1 [Reference]
Male	1.07 (1.00-1.16)	1.05 (0.97-1.14)
Race		
Black	1.03 (0.95-1.12)	1.13 (0.92-1.39)
White	0.86 (0.80-0.94)	0.98 (0.80-1.19)
Asian	1.15 (0.93-1.42)	1.20 (0.91-1.59)
Other	1.51 (0.98-2.35)	1.46 (0.91-2.34)
Unknown	1.39 (1.22-1.60)	1.15 (0.94-1.42)
Ethnicity		
Non-Hispanic	0.67 (0.60-0.75)	0.86 (0.45-1.67)
Hispanic	1.50 (1.35-1.66)	1.29 (0.67-2.50)
Unknown	1.13 (0.60-2.14)	Not included^a^
Insurance		
Public	1.15 (1.06-1.24)	0.88 (0.72-1.09)
Private	0.85 (0.78-0.92)	0.84 (0.68-1.04)
Self-pay	1.11 (0.91-1.35)	Not included^a^
No. of complex chronic conditions	1.01 (0.99-1.03)	1.02 (1.00-1.04)

^a^ Not included in the adjusted model owing to too few examples for mixed-effects coefficient estimation.

In multivariable analysis, the intervention was associated with a 3.25 (95% CI, 2.94-3.59) times higher odds of having the vaccine administered compared with historical controls (Table 2). Concurrent controls had a slightly higher odds (adjusted OR, 1.28, 95% CI, 1.15-1.42) of vaccine administration than historical controls. The week of the influenza season was also significantly associated with vaccine administration, with each additional week into the season associated with a 3.7% reduction in the odds of receiving the influenza vaccine (adjusted OR, 0.96; 95% CI, 0.96-0.97). Adolescents were less likely to receive the influenza vaccine, with 13- to 17-year-old adolescents having 28% lower odds compared with 6- to 23-month-old children (adjusted OR, 0.72; 95% CI, 0.64-0.81). No other demographic characteristics, including sex, race/ethnicity, insurance, or complex chronic conditions, were significantly associated with receipt of vaccine in the multivariable model.

### SAFER Reporting Framework

We reviewed the 8 sociotechnical dimensions of patient safety for EHR interventions and described preintervention issues, what sociotechnical changes were made, why they were felt to be effective, and how they could be applied in other settings (eTable 3 in the Supplement). The most noteworthy dimensions likely required for disseminating this intervention included the following: (1) hardware and software with an interface to the state immunization registry to improve the positive predictive value of the CDS, (2) human-computer interface using a default-checked influenza vaccine order in commonly used admission order sets, and (3) workflow and communication, where timing of the influenza vaccine order for 12 pm the day after admission improved nursing flexibility and reduced concern that vaccines may be administered without the physician team having any chance to review appropriateness.

## Discussion

This quality improvement study demonstrated that CDS was associated with increased influenza vaccine uptake among hospitalized children compared with concurrent and historical controls. The CDS was designed through analysis of a complex sociotechnical system yielding the following key features: (1) automated detection of influenza vaccine eligibility, leading to default-checked vaccine orders at admission; (2) a nursing staff script using a presumptive strategy for offering the vaccine; and (3) just-in-time education regarding appropriate contraindications and evidence-based vaccine communication strategies. These interventions stress the use of defaults as a behavioral economic strategy to influence behavior,^41,42^ including targeted defaults for clinicians to order the influenza vaccine as well as a nursing staff script emphasizing a default option for families of patients receiving the influenza vaccine during the hospitalization.

The CDS system was associated with a marked increase in the frequency of influenza vaccine orders for eligible children but with a smaller increase in actual administrations of the vaccine. This gap between orders and administrations may be due to vaccine refusal^36,43^ or other unknown barriers. Although the effect size was smaller, the influenza vaccination rate among the concurrent controls was significantly higher than among the historical controls. This difference may be due to the presumptive strategy script in the nursing admission questionnaire and communication tip sheet, which were implemented systemwide in September 2019, unlike the influenza vaccine order group, which was implemented in a stepwise fashion. It may also be due to contamination,^44^ in which the CDS led physicians to think of influenza vaccine more often even without a patient-specific EHR prompt.

Compared with prior studies of the influenza vaccine for hospitalized children, this approach aimed to reduce clinician burden by (1) automating eligibility screening as much as possible through integration with the state immunization registry, (2) providing CDS early in the workflow to maximize nursing staff flexibility for time of administration, and (3) avoiding interruptive alerts for nurses and physicians. The CDS system also did not depend on operational resources to disseminate reports or provide ongoing education. Additional benefits may be gained by combining an automated CDS approach with other evidence-based implementation science and behavioral economics interventions, such as audit and feedback, text messages to patients and families, explicit markers of vaccine intention, note template changes, and patient and family education.^6,15,45,46,47,48^ Nonetheless, scaling up these interventions would benefit from study designs that identify each bundle element to minimize the resource requirements for new sites to implement similar changes.

The implementation strategy described in this study using sequential crossover from control to intervention guided by operational constraints is practical and adds scientific rigor to CDS for quality improvement. From an operational perspective, stepwise implementations are associated with a reduced burden of change management and allow learning to be incorporated before expanding an intervention. For example, early in our first pilot on the general pediatrics service, several pharmacists noticed that influenza vaccine orders that were not administered within 12 hours of their due time would fall off the medication administration record for nurses, reducing their administration flexibility. We were able to fix this by changing the frequency setting in the EHR prior to the subsequent rollout of the intervention. From a scientific perspective, multiple comparisons with concurrent and historical controls are associated with improved internal validity of conclusions compared with simple pre-post quality improvement studies.^49,50^ Stepwise implementation guided by operational needs may still be biased because clusters that adopt an intervention early may be systematically different than later adopters, and learnings incorporated into the intervention may make the comparison of the intervention and control less clean. Stepped-wedge trials that randomize which clusters implement an intervention at each step may reduce this bias, but such trials are more costly and challenging to administer.^51^ We propose that operationally guided stepwise implementation combined with analysis comparing intervention groups with both concurrent and historical controls balances the goals of scientific rigor with operational feasibility more effectively than common pre-post designs.

### Limitations

This study has some limitations. This is a single-center study using 1 EHR from Epic Systems. Conclusions from this study may depend on organizational culture prior to the intervention, change management structures, the priority of influenza vaccination among key stakeholders, technical infrastructure, and other factors that reduce its generalizability. Although we have attempted to capture important sociotechnical elements using the SAFER Reporting Framework, these assessments are likely not comprehensive, and other factors may be associated with different outcomes when applied in other health systems. In addition, this study demonstrated the effectiveness of our intervention in only a single influenza season. The stepwise implementation was not randomized; thus, early adopters of the intervention may have had greater enthusiasm for influenza vaccination than later adopters, which could bias our results. The reasons for low influenza vaccine uptake were not systematically evaluated. Finally, in the intervention group, there remained a large fraction of patients who were not vaccinated, leaving substantial room for improvement through other evidence-based interventions.

## Conclusions

This study suggests that user-centered CDS may improve the rate of influenza vaccine administration during hospitalization for eligible children. Key elements to promote vaccine administration in acute care settings include automated determination of vaccine eligibility early in the hospitalization combined with default-checking the influenza vaccine order, providing a nursing staff script that uses a presumptive strategy to offer the vaccine, and just-in-time education regarding contraindications. Stepwise implementation of CDS interventions using sequential crossover from control to intervention is operationally feasible and improves the scientific rigor of quality improvement studies. Future studies are needed to assess whether the benefits in influenza vaccine administration are sustained across seasons, whether similar results are seen when applied to other institutions, and whether this intervention model can scale to other health maintenance interventions in acute care settings. Despite the improvements in influenza vaccine uptake demonstrated in this study, there remains a substantial gap between patients eligible for influenza vaccine and actual administrations, even in the intervention group. Additional work to understand persistent reasons for low uptake and the potential effect of combining CDS with other behavioral economic and implementation science interventions would likely reduce the burden of influenza in a vulnerable population and provide lessons to improve vaccine coverage for other diseases, such as COVID-19.

## Notes

Supplement. **eTable 1.** Preintervention User and Task Analysis for Influenza Vaccine Administration, Associated Barriers, and Interventions **eTable 2.** Phased Implementation of the Dynamic Influenza Vaccine Order Group by Order Set **eTable 3.** Sociotechnical Interventions Described Using the SAFER Reporting Framework for Safety Related EHR Research **eFigure 1.** Preintervention Discharge Navigator Non-Interruptive Alert Developed for 2018-2019 Influenza Season **eFigure 2.** Clinical Decision Support System to Promote Influenza Vaccine in Hospitalized Children
