# Supplementary material for: Evaluation of a Clinical Decision Support Strategy to Increase Seasonal Influenza Vaccination Among Hospitalized Children Before Inpatient Discharge
Source: JAMA Netw Open. 2021 Jul 22;4(7):e2117809. doi: 10.1001/jamanetworkopen.2021.17809 (PMC8299313; doi:10.1001/jamanetworkopen.2021.17809)
Supplement: Supplement. — eTable 1. Preintervention User and Task Analysis for Influenza Vaccine Administration, Associated Barriers, and Interventions eTable 2. Phased Implementation of the Dynamic Influenza Vaccine Order Group by Order Set eTable 3. Sociotechnical Interventions Described Using the SAFER Reporting Framework for Safety Related EHR Research eFigure 1. Preintervention Discharge Navigator Non-Interruptive Alert Developed for 2018-2019 Influenza Season eFigure 2. Clinical Decision Support System to Promote Influenza Vaccine in Hospitalized Children [file jamanetwopen-e2117809-s001.pdf]

Supplementary Online Content

Orenstein EW, ElSayed-Ali O, Kandaswamy S, et al. Evaluation of a clinical decision support strategy to increase seasonal influenza vaccination among hospitalized children before inpatient discharge. *JAMA Netw Open.* 2021;4(7):e2117809. doi:10.1001/jamanetworkopen.2021.17809

eTable 1. Preintervention User and Task Analysis for Influenza Vaccine Administration, Associated Barriers, and Interventions

eTable 2. Phased Implementation of the Dynamic Influenza Vaccine Order Group by Order Set

eTable 3. Sociotechnical Interventions Described Using the SAFER Reporting Framework for Safety Related EHR Research

eFigure 1. Preintervention Discharge Navigator Non-Interruptive Alert Developed for 2018-2019 Influenza Season

eFigure 2. Clinical Decision Support System to Promote Influenza Vaccine in Hospitalized Children

This supplementary material has been provided by the authors to give readers additional information about their work.

© 2021 Orenstein EW et al. *JAMA Network Open.*

eTable 1. Preintervention User and Task Analysis for Influenza Vaccine Administration, Associated Barriers, and Interventions

Task	User	Barrier(s)	Intervention(s)
Determine eligibility	Physician	• Multiple clicks to review immunizations.<br>• Manual review of immunizations to determine eligibility.<br>• Incomplete capture in immunization registry.<br>• Misunderstanding of contraindications to vaccination.	• System automatically identifies patients ≥6 months with no influenza vaccine in current season.<br>• Nursing admission questionnaire captures immunizations not present in state registry.<br>• Influenza vaccine order display addresses common misunderstandings.
Obtain verbal consent	Physician	• Time to address concerns for families hesitant to give influenza vaccine.<br>• Knowledge of vaccine facts and evidence-based communication strategies.	• Opt out wording for influenza vaccine in nursing admission questionnaire.<br>• Communication tip-sheet integrated into influenza vaccine order.
Order Vaccine	Physician	• Remember to address influenza vaccine even if not related to chief complaint.	• Influenza vaccine automatically added to admission order sets when eligibility criteria met.
Allocate and transport vaccine	Pharmacy	• Family unwilling to wait for vaccine when close to discharge.	• Prompt for influenza vaccine in admission order sets, earlier in workflow than time of discharge.
Administer vaccine	Nurse	• Family refusal after initial consent.<br>• Missed in context of many other discharge tasks.	• Prompt for influenza vaccine in admission order sets, earlier in workflow than time of discharge.
Document vaccine	Nurse	• Ambulatory and inpatient documentation requirements differ (e.g. if patient is VFC eligible)	• Reduce required fields in vaccine documentation.

VFC: Vaccines for Children

© 2021 Orenstein EW et al. *JAMA Network Open.*

eTable 2. Phased Implementation of the Dynamic Influenza Vaccine Order Group by Order Set

**Order Set Name**	**Phase 1 (9/19/2019)** *Hospital A Only*	**Phase 2A (10/3/2019)** *Hospitals A & B Only*	**Phase 2B (10/29/2019)** *Hospitals A & B Only*	**Phase 3 (11/19/2019)** *Hospitals A, B, and C*
Phase 1				
PED General Admission	X	X	X	X
Phase 2A				
HEM Admit New Bleeding Patient		X	X	X
HEM Anemia Admission		X	X	X
HEM Sickle Cell Disease Admission		X	X	X
HEM Thrombosis Admission		X	X	X
HEM/ONC General Admission Orders		X	X	X
NEU VEEG (EMU) Admission		X	X	X
NEU Video Epilepsy Surgery Eval		X	X	X
ONC Fever/Neutropenia for Est Oncology Patient Admission		X	X	X
ONC New Leukemia/Pancytopenia Admission		X	X	X
ONC New Solid Tumor Admission		X	X	X
PED Abdominal Pain Admission		X	X	X
PED Bites (Human or Animal) Admission		X	X	X
PED Cellulitis/Abscess/Soft Tissue Infection Admission		X	X	X
PED Failure to Thrive Admission		X	X	X
PED Henoch-Schonlein Purpura with Abdominal Pain Admission		X	X	X
PED Inpatient Asthma Guideline		X	X	X
PED Kawasaki Admission		X	X	X
PED Lymphadenopathy/Lymphadenitis Admission		X	X	X
PED Non-Accidental Trauma Admission		X	X	X
PED Periorbital/Orbital Cellulitis Admission		X	X	X
PED Rhabdomyolysis Admission		X	X	X
PED Tylenol Ingestion Admission		X	X	X

© 2021 Orenstein EW et al. *JAMA Network Open.*

Order Set Name	**Phase 1 (9/19/2019)** *Hospital A Only*	**Phase 2A (10/3/2019)** *Hospitals A & B Only*	**Phase 2B (10/29/2019)** *Hospitals A & B Only*	**Phase 3 (11/19/2019)** *Hospitals A, B, and C*
PED Urinary Tract Infection – Complicated Admission		X	X	X
PED Suicide Admission		X	X	X
PICU General Admission – EG		X	X	X
PICU Status Asthmaticus Admission		X	X	X
PICU Status Epilepticus Admission		X	X	X
Phase 2B				
GI Abdominal Pain Admission			X	X
GI Cholangitis Admission			X	X
GI Cholestasis Admission			X	X
GI CVL Sepsis Admission			X	X
GI Cyclic Vomiting Admission			X	X
GI Failure to Thrive Admission			X	X
GI General Admission			X	X
GI Inflammatory Bowel Flare Up Admission			X	X
GI Nasogastric Cleanout Admission			X	X
GI Pancreatitis Admission			X	X
GI Remicade Administration			X	X
NEO General Admission – SR			X	X
ONC Discharge Orders			X	X
PUL Cystic Fibrosis Exacerbation Admission			X	X
PUL ICU Trach-Vent Admission			X	X
PUL Respiratory Distress Admission			X	X
Phase 3				
HS IP Asthma Guideline Admission				X
HS IP Bronchiolitis Guideline Admission				X
HS IP Croup Guideline Admission				X
HS IP PED Abdominal Pain Admission				X
HS IP PED AGE-Needs Fluids Admission				X
HS IP PED Cellulitis/Abscess/Soft Tissue Infection Admission				X

© 2021 Orenstein EW et al. *JAMA Network Open.*

Order Set Name	**Phase 1 (9/19/2019)** *Hospital A Only*	**Phase 2A (10/3/2019)** *Hospitals A & B Only*	**Phase 2B (10/29/2019)** *Hospitals A & B Only*	**Phase 3 (11/19/2019)** *Hospitals A, B, and C*
HS IP PED Concussion/Mild Head Injury Admission				X
HS IP PED Failure to Thrive Admission				X
HS IP PED General Admission				X
HS IP PED Sickle Cell Disease Admission				X
IP Diabetic Ketoacidosis (DKA) Guideline Order Set				X
REN Nephrotic Syndrome Admission				X

© 2021 Orenstein EW et al. *JAMA Network Open.*

eTable 3. Sociotechnical Interventions Described Using the SAFER Reporting Framework for Safety Related EHR Research[27]

Sociotechnical Dimension	Pre-Intervention Issues	What*	Why†	How‡
**Hardware & Software**	EHR vendor allows interface with state immunization registry.	• Created rule to evaluate if influenza vaccine present in state registry in current influenza season.<br>• Ensured query to state registry occurs automatically at start of encounter.	Integration with registry increases likelihood that patient is genuinely eligible for influenza vaccine when system suggests it. Higher PPV improves trust.	• EHR integration with state immunization registries.<br>• Validate rules to determine if influenza vaccine given in current season.
**Clinical Content**	• Common misconceptions of vaccine contraindications (e.g. patient on steroids, immunocompromised).<br>• Lack of awareness of evidence-based vaccine communication strategies.	• Guidance in influenza vaccine order indicating that vaccine is safe and recommended in key population with links out to supporting literature.<br>• Communication tip sheet into influenza vaccine order.<br>• Nursing admission questionnaire uses opt-out strategy when asking about influenza vaccine.	Supporting literature and guidance at right time in workflow provides just-in-time education.	• Identify local knowledge gaps.<br>• Provide appropriate educational resources in influenza vaccine order.<br>• Use opt-out scripts when asking about influenza vaccine.
**Human-Computer Interface**	Prompt for influenza vaccine order very late in the workflow, easily ignored.	Default-checked influenza vaccine order in admission order sets when eligible.	Defaults are powerful behavioral nudges.	• Use rules to automatically identify patients eligible for influenza vaccine.<br>• Use default or standing vaccine orders when criteria are met.
**Workflow & Communication**	• Nurse collection of immunization information at admission not connected to physician ordering.	• Nursing documentation of families who already received influenza vaccine or who refuse vaccine suppresses default-checked physician order.	• Incorporating nursing documentation into rules increases likelihood that patient is genuinely eligible for influenza vaccine when	• Integrate state immunization registry and nursing documentation to recommend standing or

© 2021 Orenstein EW et al. *JAMA Network Open.*

Sociotechnical Dimension	Pre-Intervention Issues	What*	Why†	How‡
	• Time of discharge very hectic; vaccines ordered at that time often missed or family refuses to wait for vaccine.<br>• Concerns for wasting vaccines if allocated by pharmacy but then refused by family.	• Decision support recommends influenza vaccine at 12pm on day after admission instead of discharge.<br>• Nurse calls pharmacy to obtain influenza vaccine after confirming consent with family and timing.	system suggests it. Higher PPV improves trust.<br>• Suggested timing of vaccine allows physicians to round on patient prior to administration, but maximizes nursing flexibility for when to give the vaccine.	default-checked influenza vaccine order.<br>• Time influenza vaccine order for shortly after admission (to maximize nursing flexibility) instead of discharge.
**People**	Concerns about inappropriate influenza vaccine administration, timing, or parental reactions.	Quasi-experimental implementation of default influenza vaccine orders in admission order sets after approval of order set clinical owners.	Increased trust from more rigorous evaluation. Opportunity for concerns to be addressed early in a pilot phase prior to expansion.	Consider staged implementation to ensure success locally.
**Internal Organizational Features**	• Presence of quality improvement, clinical informatics, data analytics, and human factors engineering expertise within the organization.<br>• Strong culture promoting influenza vaccines in leadership and front-line staff.	• Early advocacy for analysis, user-centered design, and quasi-experimental implementation approach.<br>• Created feedback loop to ensure organizational leadership aware of problem investigation, interventions, measurement framework, and results.	Data-driven intervention and implementation design facilitated buy-in from concerned stakeholders.	• Obtain executive sponsorship early.<br>• Develop automated queries to detect changes in influenza vaccine uptake early.
**External Rules & Regulations**	•Vaccine reimbursement limited in inpatient as hospital was not a Vaccines For Children site.<br>• External benchmarking (e.g. USNWR) interested in influenza vaccination	• Discussed with hospital finance teams potential for poor reimbursement.<br>• Executive sponsorship to perform pilot evaluation and work to become Vaccines For Children site.	Clinical operations able to prioritize public health and hospital rankings goals over immediate financial goals.	Engage health system leaders early and promote benefits to public health and hospital rankings.

© 2021 Orenstein EW et al. *JAMA Network Open.*

Sociotechnical Dimension	Pre-Intervention Issues	What*	Why†	How‡
	rates in vulnerable populations.	• Advocacy for potential to improve USNWR scores.		
**Measuring & Monitoring**	• Existing reporting framework for influenza vaccine rates using state registry data.<br>• Reports did not have ability to exclude patients screened out by nursing or to report based on order sets used.	• Created automated reports to identify (1) all hospitalizations eligible for influenza vaccine, (2) screened out by nursing, (3) influenza vaccine orders and administrations, and (4) order sets used in the hospitalization.<br>• Leveraged existing reports for balance measures.	Automated reports facilitated rapid PDSA cycles and expansion of the intervention.	Create background alert for patients eligible for influenza vaccine to inform subsequent reporting.

EHR: electronic health record; IT: Information Technology; VS: vital signs;

* What sociotechnical changes were made to implement an EHR-related intervention to improve patient safety.

† Why the intervention did or did not lead to safety improvements.

‡ How the intervention can be applicable or exported to others.

© 2021 Orenstein EW et al. *JAMA Network Open.*

eFigure 1. Preintervention Discharge Navigator Non-Interruptive Alert Developed for 2018-2019 Influenza Season

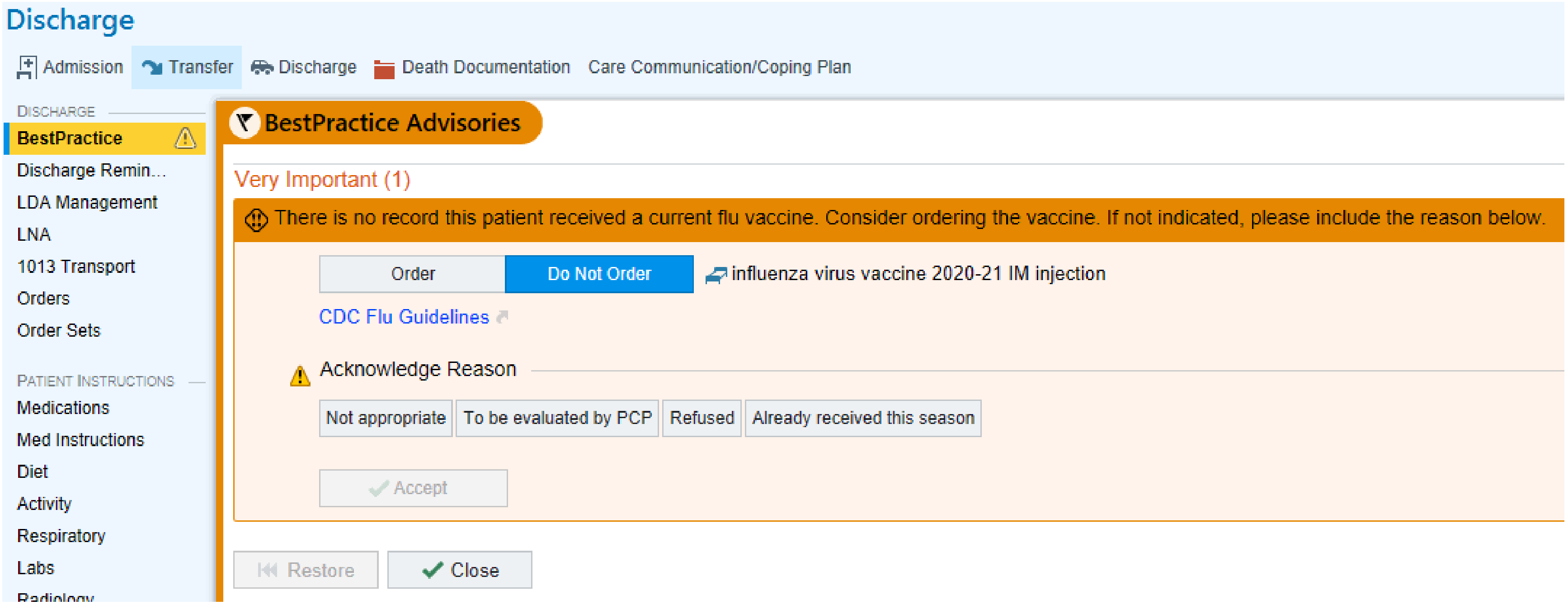

© 2021 Orenstein EW et al. *JAMA Network Open.*

eFigure 2. Clinical Decision Support System to Promote Influenza Vaccine in Hospitalized Children

2A: Nursing Admission Questionnaire

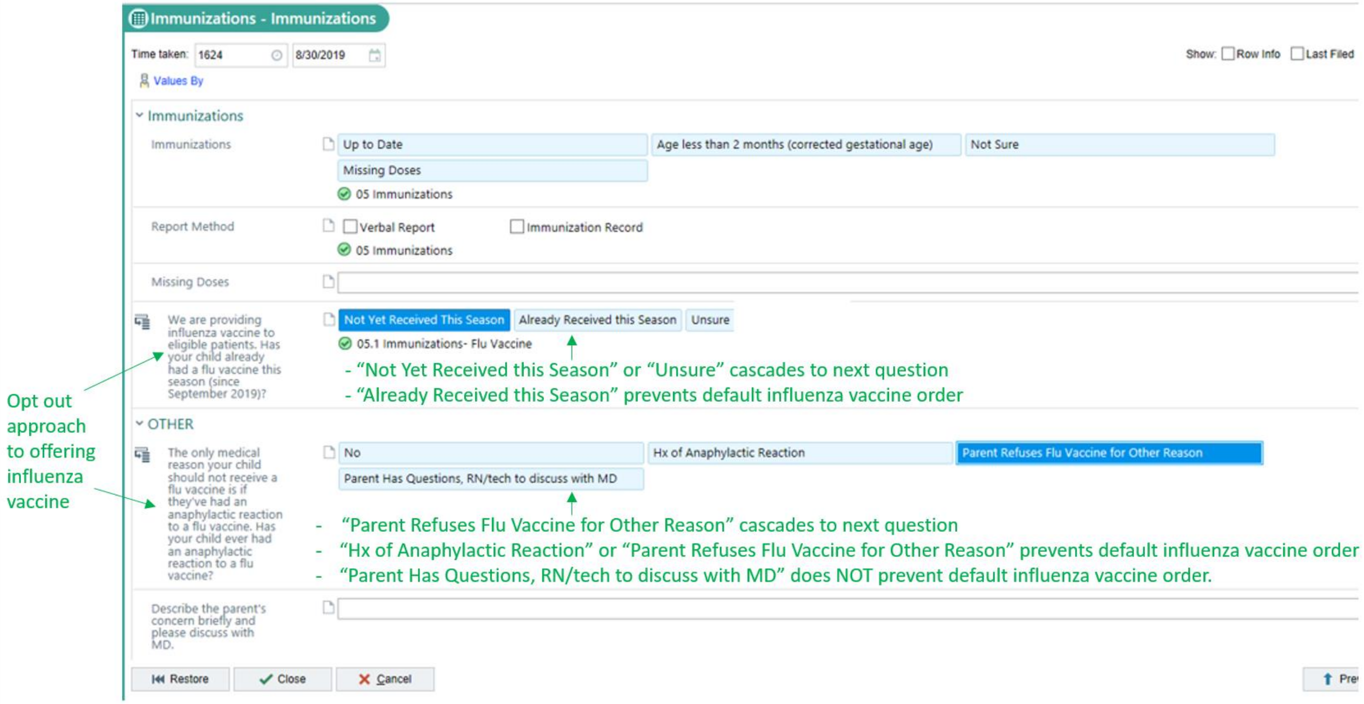

2B: Influenza Vaccine Order Group

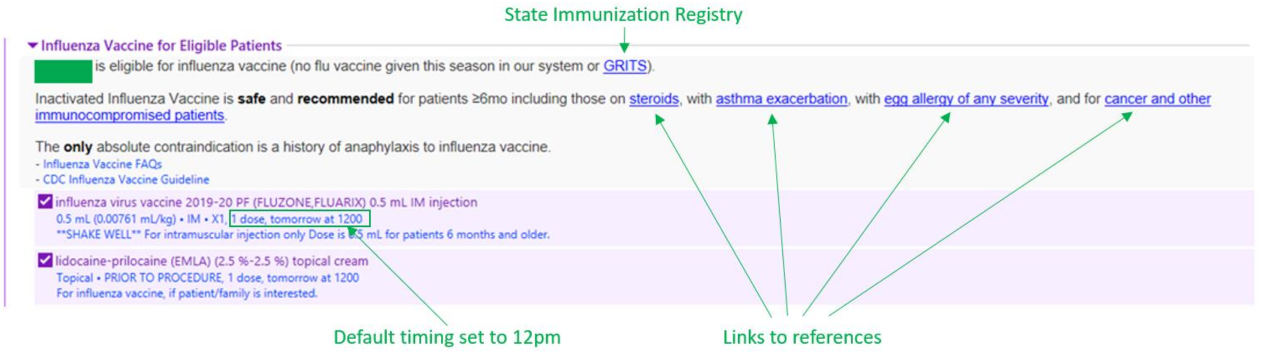

© 2021 Orenstein EW et al. *JAMA Network Open.*
